# Supplementary material for: From sequence to enzyme mechanism using multi-label machine learning
Source: BMC Bioinformatics. 2014 May 19;15:150. doi: 10.1186/1471-2105-15-150 (PMC4229970; doi:10.1186/1471-2105-15-150)
Supplement: Additional file 2 — Java code of ml2db. Additional file ml2db_code.tar.gz contains the Java source code to run the multi-label machine learning experiments and save the results to database. The code’s Javadoc is included. [file 1471-2105-15-150-S2.zip › additional file 2/ml2db/ml2db/doc/mulan/data/GreedyLabelClustering.html]

GreedyLabelClustering


JavaScript is disabled on your browser.


- Overview
- Package
- Class
- Use
- Tree
- Deprecated
- Index
- Help

- Prev Class
- Next Class

- Frames
- No Frames

- All Classes

- Summary:
- Nested |
- Field |
- Constr |
- Method

- Detail:
- Field |
- Constr |
- Method


mulan.data

## Class GreedyLabelClustering

- java.lang.Object
- - mulan.data.GreedyLabelClustering

- All Implemented Interfaces:
  :   java.io.Serializable, LabelClustering

  ---

    

  ```
  public class GreedyLabelClustering
  extends java.lang.Object
  implements LabelClustering, java.io.Serializable
  ```

  A class for clustering dependent label pairs into disjoint subsets.   
    
  The type of the learned dependencies is determined by the
  `LabelPairsDependenceIdentifier` supplied to the class constructor. The
  clustering process is straightforward: initially all labels are assumed to be independent. Then
  we start group the label pairs according to their dependence score from most to least dependent.
  An SubsetLearner is build for each new partition and its accuracy is evaluated in terms of the
  `measure`. The process of grouping labels continues as long as the accuracy improves (or
  at least is not reduced). A number of steps specified by `allowedNonImprovementSteps`
  without seeking any concomitant improvement in the accuracy is allowed. Such a �non-useful�
  partitions are filtered out and the algorithm continues to evaluate subsequent pairs of dependent
  labels until one of the stop conditions is reached. The possible stop conditions are:   
  - no more label pairs to consider;   
  - all labels are clustered into one single group;   
  - pair dependence score is below the specified `criticalValue`;   
  - the number of `allowedNonImprovementSteps` is exceeded.

  Version:
  :   05.05.2011

  Author:
  :   Lena Chekina (lenat@bgu.ac.il)

  See Also:
  :   Serialized Form

- - ### Constructor Summary

    Constructors

    Constructor and Description

    `GreedyLabelClustering(MultiLabelLearner aMultiLabelLearner,
    weka.classifiers.Classifier aSingleLabelLearner,
    LabelPairsDependenceIdentifier dependenceIdentifier)`

    Initialize the GreedyLabelClustering with multilabel and single label learners and a method
    for labels dependence identification.
  - ### Method Summary

    Methods

    | Modifier and Type | Method and Description |
    | --- | --- |
    | `int[][]` | `determineClusters(MultiLabelInstances trainingSet)` Determines labels partitioning into dependent sets. |
    | `int` | `getAllowedNonImprovementSteps()` |
    | `double` | `getCriticalValue()` |
    | `Measure` | `getMeasure()` |
    | `MultiLabelLearner` | `getMultiLabelLearner()` |
    | `int` | `getNumFolds()` |
    | `weka.classifiers.Classifier` | `getSingleLabelLearner()` |
    | `boolean` | `isInternalSubsetLearnerDebug()` |
    | `boolean` | `isUseSubsetLearnerCache()` |
    | `static java.lang.String` | `partitionToString(int[][] partition)` Returns a string representation of the labels partition. |
    | `void` | `setAllowedNonImprovementSteps(int allowedNonImprovementSteps)` |
    | `void` | `setCriticalValue(double criticalValue)` |
    | `void` | `setInternalSubsetLearnerDebug(boolean internalSubsetLearnerDebug)` |
    | `void` | `setMeasure(Measure measure)` |
    | `void` | `setNumFolds(int numFolds)` |
    | `void` | `setUseSubsetLearnerCache(boolean useSubsetLearnerCache)` |

    - ### Methods inherited from class java.lang.Object

      `equals, getClass, hashCode, notify, notifyAll, toString, wait, wait, wait`

- - ### Constructor Detail


    - #### GreedyLabelClustering

      ```
      public GreedyLabelClustering(MultiLabelLearner aMultiLabelLearner,
                           weka.classifiers.Classifier aSingleLabelLearner,
                           LabelPairsDependenceIdentifier dependenceIdentifier)
      ```

      Initialize the GreedyLabelClustering with multilabel and single label learners and a method
      for labels dependence identification.

      Parameters:
      :   `aMultiLabelLearner` - - a learner for multilabel classification
      :   `aSingleLabelLearner` - - a learner for single label classification
      :   `dependenceIdentifier` - - a method for label pairs dependence identification
  - ### Method Detail


    - #### determineClusters

      ```
      public int[][] determineClusters(MultiLabelInstances trainingSet)
      ```

      Determines labels partitioning into dependent sets. It clusters label pairs according to
      their dependence score and evaluates the related models. The clustering process continues as
      long as the accuracy improves. The finally selected labels partition is returned.

      **Specified by:**
      :   `determineClusters` in interface `LabelClustering`

      Parameters:
      :   `trainingSet` - the training data set

      Returns:
      :   a label set partitioning


    - #### partitionToString

      ```
      public static java.lang.String partitionToString(int[][] partition)
      ```

      Returns a string representation of the labels partition.

      Parameters:
      :   `partition` - - a label set partition

      Returns:
      :   a string representation of the labels partition


    - #### getNumFolds

      ```
      public int getNumFolds()
      ```

      Returns:


    - #### setNumFolds

      ```
      public void setNumFolds(int numFolds)
      ```

      Parameters:
      :   `numFolds` -


    - #### getMeasure

      ```
      public Measure getMeasure()
      ```

      Returns:


    - #### setMeasure

      ```
      public void setMeasure(Measure measure)
      ```

      Parameters:
      :   `measure` -


    - #### getAllowedNonImprovementSteps

      ```
      public int getAllowedNonImprovementSteps()
      ```

      Returns:


    - #### setAllowedNonImprovementSteps

      ```
      public void setAllowedNonImprovementSteps(int allowedNonImprovementSteps)
      ```

      Parameters:
      :   `allowedNonImprovementSteps` -


    - #### getCriticalValue

      ```
      public double getCriticalValue()
      ```

      Returns:


    - #### setCriticalValue

      ```
      public void setCriticalValue(double criticalValue)
      ```

      Parameters:
      :   `criticalValue` -


    - #### getSingleLabelLearner

      ```
      public weka.classifiers.Classifier getSingleLabelLearner()
      ```

      Returns:


    - #### getMultiLabelLearner

      ```
      public MultiLabelLearner getMultiLabelLearner()
      ```

      Returns:


    - #### isUseSubsetLearnerCache

      ```
      public boolean isUseSubsetLearnerCache()
      ```

      Returns:


    - #### setUseSubsetLearnerCache

      ```
      public void setUseSubsetLearnerCache(boolean useSubsetLearnerCache)
      ```

      Parameters:
      :   `useSubsetLearnerCache` -


    - #### isInternalSubsetLearnerDebug

      ```
      public boolean isInternalSubsetLearnerDebug()
      ```

      Returns:


    - #### setInternalSubsetLearnerDebug

      ```
      public void setInternalSubsetLearnerDebug(boolean internalSubsetLearnerDebug)
      ```

      Parameters:
      :   `internalSubsetLearnerDebug` -


- Overview
- Package
- Class
- Use
- Tree
- Deprecated
- Index
- Help

- Prev Class
- Next Class

- Frames
- No Frames

- All Classes

- Summary:
- Nested |
- Field |
- Constr |
- Method

- Detail:
- Field |
- Constr |
- Method
